# Folliculocentric lymphocytic hypersensitivity reactions in CLL/SLL patients: A unique clinicopathologic entity amongst non‐specific hypersensitivity reactions

**DOI:** 10.1002/ski2.208

**Published:** 2023-01-19

**Authors:** James Abbott, Jessica Corean, Ashley M. Snyder, Scott R. Florell, Rodney Miles, Deborah Stephens, David A. Wada

**Affiliations:** ^1^ Department of Dermatology University of Utah Salt Lake City Utah USA; ^2^ Department of Pathology University of Utah Salt Lake City Utah USA; ^3^ Department of Population Health Sciences University of Utah Salt Lake City Utah USA; ^4^ Huntsman Cancer Institute Salt Lake City Utah USA; ^5^ Department of Hematology and Hematologic Malignancies University of Utah Salt Lake City Utah USA

## Abstract

**Background:**

Cutaneous hypersensitivity eruptions in chronic lymphocytic leukemia (CLL)/small lymphocytic lymphoma (SLL) are a clinically and histologically heterogeneous group that can either precede, occur with, or follow the development of a hematologic malignancy. Therefore, establishing the diagnosis requires careful clinical and pathologic correlation and an understanding of the broad spectrum of presentations. Data is lacking on the correlation of skin disease with molecular/cytogenetic risk profiling of the tumor.

**Objectives:**

The aims of this study were to characterize the clinical, histological, and genetic aberrations in recurrent cutaneous hypersensitivity reactions in patients with CLL/SLL.

**Methods:**

A single site academic retrospective chart review of medical records, histopathology, molecular and cytogenetic data in CLL/SLL patients who developed biopsy‐proven cutaneous hypersensitivity reactions.

**Results:**

Five hundred one new diagnoses of CLL/SLL with 73 patients requiring cutaneous biopsies for skin lesions or rashes were identified. With exclusion criteria, 20 biopsies were identified from 17 patients (mean age, 69.6 years, females = 9) with unexplained cutaneous eruptions. These were commonly pruritic, erythematous papules above the waist. Most biopsies had a prominent superficial, deep dermal eosinophilic infiltrate (85%), with a robust T‐cell predominant dermal infiltrate in 40%. Five out of 17 patients (29%) had a predominately folliculocentric CD4+ T‐cell infiltrate; all occurring on the head and neck. Overall, the prevalence of cutaneous hypersensitivity eruptions requiring biopsy was 3.4% (*n* = 17), and the prevalence of folliculocentric CD4+ T‐cell infiltrate was 1% (*n* = 5).

**Conclusion:**

Cutaneous hypersensitivity reactions in CLL/SLL are heterogeneous; however, folliculotropic CD4+ T‐cell infiltrates may be seen in a small but distinct clinical subset of patients. Commonly tested cytogenetic aberrations in CLL/SLL do not appear to be correlated with the presence of cutaneous hypersensitivity reactions.

## Graphical abstract

Hypersensitivity reactions are common in CLL/SLL patients with CD4+ folliculocentric infiltrates being a potential specific clinicopathologic entity. These hypersensitivity reactions do not appear to be increased in the commonly tested genetic studies for CLL/SLL. Treatment of the underlying lymphoma may alleviate hypersensitivity reactions.

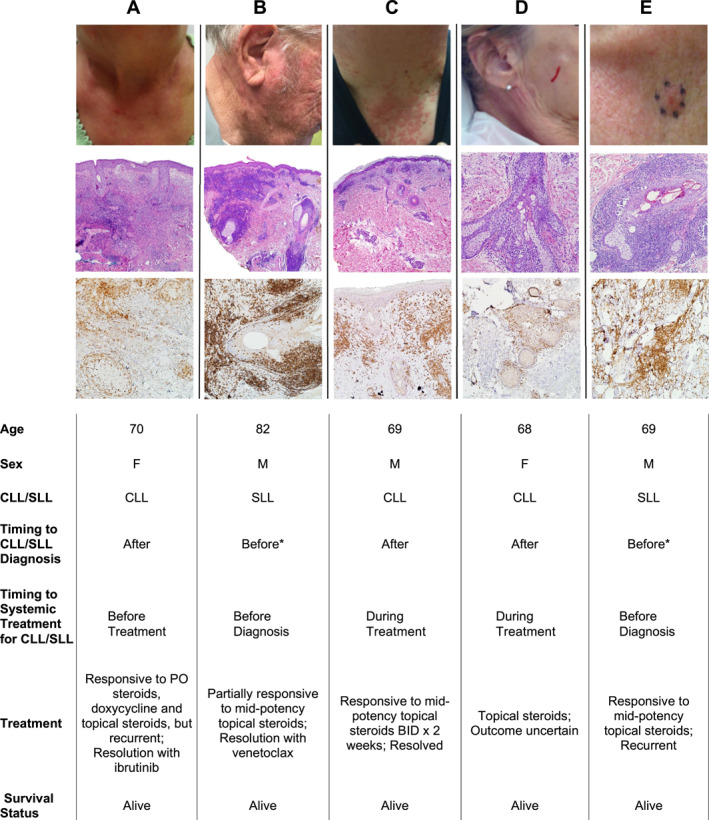

1

**What is already known about this topic?**

- Hypersensitivity reactions in chronic lymphocytic leukemia (CLL)/small lymphocytic lymphoma (SLL) patients represent a diverse array of dermatoses that can be robust and significantly impact patients' quality of life due to symptoms and lack of effective treatment. Although both clinically and histologically heterogeneous, there have been attempts to characterize these eruptions based on specific histological patterns: insect‐bite‐like reactions, eosinophilic dermatosis of hematologic malignancy (EDHM), and most recently as T‐Cell Papulosis of B‐cell Malignancy (TCP‐BCM). Despite these efforts, the diagnosis of paraneoplastic hypersensitivity reactions remains challenging due to the lack of specific histological findings.

**What does this study add?**

- We describe a cohort of CLL/SLL patients with a prominent CD4+ folliculocentric infiltrate that have a characteristic clinicopathologic findings. These findings corroborate a prior study by Visseaux et al. Additionally, we show that commonly tested genetic aberrations in CLL/SLL do not appear to be increased in these hypersensitivity reactions (i.e. more aggressive lymphomas may not be associated with more aggressive cutaneous hypersensitivity reactions). Finally we illustrate that several patients with recurrent cutaneous involvement had their cutaneous disease resolve with treatment of the underlying CLL/SLL.

## 2 INTRODUCTION

Chronic lymphocytic leukemia (CLL) and its predominantly nodal counterpart, termed small lymphocytic lymphoma (SLL), is the most prevalent adult leukemia in the US, with an estimated 190 000 people living with disease.^1^ Cutaneous manifestations are well‐documented in CLL/SLL and represent a diverse group of inflammatory and neoplastic disorders. This finding is likely related to impaired immune competence from the underlying systemic leukemia/lymphoma. In some situations, neoplastic B lymphocytes can be found within the skin as co‐inhabitants due to the reactive phenomena, as a principle infiltrate (leukemia cutis), or as an underlying driver for a cutaneous eruption.^2^, ^3^ Notably, when diagnosing paraneoplastic inflammatory dermatoses associated with CLL/SLL, leukemia cutis should be excluded by definition.

Hypersensitivity reactions in CLL/SLL patients represent a diverse array of dermatoses that can be robust and significantly impact patients' quality of life due to symptoms and lack of effective treatment. Although both clinically and histologically heterogeneous, there have been attempts to characterize these eruptions based on specific histological patterns: insect‐bite‐like reactions, EDHM, and most recently as TCP‐BCM.^3^, ^4^, ^5^, ^6^, ^7^ Despite these efforts, the diagnosis of paraneoplastic hypersensitivity reactions remains challenging due to the lack of specific histological findings.

Herein, we present our findings from a review of our institutional databases to clinically identify cutaneous hypersensitivity reactions in CLL/SLL and describe their associated histologic spectrum. The study objectives were to identify recurrent histological and clinical patterns and their relationship to the underlying CLL/SLL. Additionally, molecular and cytogenetic characteristics of the underlying CLL/SLL were reviewed to determine if there was a correlation between risk profiles and the presence of cutaneous hypersensitivity reactions.

## 3 MATERIAL AND METHODS

A single‐center retrospective study was conducted at the University of Utah using institutional electronic medical records databases from 2010 to 2019. Patients were captured through the Department of Dermatology by identifying ICD‐9 and −10 codes 204.10, C91.1 [CLL], 202.80, and C83.0 [SLL] and CPT codes 11100 and 11101 for biopsies. Inclusion criteria for thorough review included a confirmed diagnosis of CLL or SLL, biopsy of a cutaneous eruption not explained by another process, and clinical follow‐up in the Department of Dermatology. Biopsies were reviewed only if a reactive inflammatory infiltrate was described. Patients with vesiculobullous eruptions must have had negative or non‐diagnostic direct immunofluorescence (DIF) findings to exclude bullous pemphigoid. Patients with the following criteria were excluded: other (non‐CLL/SLL) hematologic malignancies that did not represent a transformation of CLL/SLL and absence of biopsy or clinical‐histological findings consistent with a specific dermatological diagnosis (i.e., infections, common urticaria). Cutaneous neoplasms and leukemia/lymphoma cutis were also excluded from this cohort. Available clinical, photographic, and histological data were reviewed. Seasonality was based on the North American calendar. A panel of dermatopathologists reviewed skin biopsies with hematoxylin and eosin (H&E), immunohistochemical (IHC), and special stains. IHC and special stains, including CD3, CD4, CD5, CD8, CD20, periodic acid‐Schiff, and Gram stains, were reviewed when provided. Histological findings were characterized based on epidermal and dermal changes with a characterization of the inflammatory infiltrate according to the prevailing histological patterns. Complete blood counts (CBC) were reviewed and recorded if studies were performed at the time of skin biopsy. Immunoglobulin heavy chain variable (IgHV) mutation analysis by sequencing and fluorescence in situ hybridisation (FISH)z panel for ATM (11q22.3), chromosome 12 centromere, D135319 (13q14.3), and p53 (17p13.1) were reviewed in select patients.

## 4 RESULTS

Of 501 total CLL/SLL patients diagnosed from 2010 to 2019 at the University of Utah and Huntsman Cancer Institute, 73 patients had biopsies in the Department of Dermatology from 2010 to 2019 for further evaluation of cutaneous lesions or dermatoses. Most biopsies performed were for cutaneous carcinomas. After applying inclusion and exclusion criteria, 17 patients with 20 biopsies were identified. Thus, the prevalence of non‐specific hypersensitivity cutaneous eruptions (*n* = 17) requiring biopsy in CLL/SLL patients between 2010 and 2019 was 3.4%.

### 4.1 Demographics and clinical characteristics (Table 1)

Of the 17 patients included for thorough review, our cohort was composed of a primarily elderly demographic with an approximately equal distribution of males to females. On average, cutaneous eruptions were present 11 months before biopsy, ranging from 2 weeks to 5 years. Clinically, these cutaneous eruptions were heterogeneous and were seen relatively uniform across the body as scattered erythematous papules or plaques. Pruritus was the leading complaint in 71% of patients. A minority of patients were asymptomatic (24%). In most patients, summer (45%) was the most frequent season when lesions were first biopsied, although cutaneous eruptions spanned several seasons before a biopsy.

**TABLE 1:** Summary of demographic and clinical findings

Demographics	*n* = 17	(%)
Age (mean)	69.6	
Sex (M:F)	8:9	

Duration of lesions prior to biopsy (months)		
Mean	10.9	
Median	4	
Range	0.5–60	

Location of lesions		
Head/Neck	9	53
Chest	10	59
Back	8	47
Upper extremities	10	59
Hands	1	6
Lower extremities	8	47

Clinical morphology		
Papule	15	88
Plaque	9	53
Nodule	2	12
Vesiculobullous	3	18
Urticarial	2	12
Eczematous	5	29
Erosions	6	35
Purpuric	3	18
Edematous	2	12

Symptoms		
Pruritus	12	71
Burning	2	12
Pain	1	6
None	4	24
Unknown	1	6

Season at time of skin biopsy	n = 20	
Summer	9	45
Fall	2	10
Winter	4	20
Spring	5	25

*Note*: Pruritic papules were seen with relative uniformity throughout the body. Multiple values for location and morphology were recorded for each patient based on clinical documentation, reflecting the extent and heterogeneity of these cutaneous eruptions.

### 4.2 CLL/SLL characteristics (Table 2)

Most patients had a CLL diagnosis (88%) based on peripheral blood involvement, with only two patients having SLL diagnosed on lymph node biopsy. At the time of skin biopsy, lymphadenopathy (62%) was the most common symptom of the underlying hematopoietic malignancy. FISH, IgHV, and CBC (at cutaneous biopsy) were reviewed in those who had these studies performed for prognostic purposes. 13q14 deletion was the most common cytogenetic aberration, seen in half the patients. CBC at the time of cutaneous biopsy revealed that most patients had an elevated WBC (reference range, 4.30–11.30 k/ul) with lymphocytosis as the predominant abnormality on the differential. Seventy‐one percent of patients had a diagnosis of CLL/SLL before developing a cutaneous eruption; however, 30% of patients either had a cutaneous eruption prior or concurrently to a diagnosis of CLL/SLL. The cutaneous eruption prompted an investigation into an underlying hematologic malignancy in two patients. Eleven patients (65%) had cutaneous eruptions before initiating systemic treatment for CLL/SLL, and treatment was initiated 5 years (mean) after the diagnosis of CLL/SLL in our cohort. Treatment modalities for CLL/SLL were highly variable, with most patients receiving multiple agents throughout their care. At the time of manuscript preparation, 82% of patients were alive.

**TABLE 2:** Characteristics of chronic lymphocytic leukemia (CLL) and small lymphocytic lymphoma (SLL) and their temporal relationship to biopsies for cutaneous

Leukemia/Lymphoma characteristics	*n* = 17	(%)
CLL*	15	88
SLL	2	12

CLL/SLL symptoms at skin biopsy	*n* = 13	
Anemia	6	46
Lymphadenopathy	8	62
Splenomegaly	5	38
Fatigue	6	46

Cytogenetics and molecular	*n* = 14	
IgHV (*n* = 8)		
Mutated	3	38
Unmutated	5	63
Cytogenetics		
11q22 deletion	4	29
13q14 deletion	7	50
17p13 deletion	1	7
Trisomy 12	4	29

First cutaneous eruptions occurrence to patient diagnosis of CLL/SLL	*n* = 17	
Before DX	3	18
Concurrent	2	12
After DX	12	71

Latency of cutaneous eruption after diagnosis (years)	*n* = 12
Mean	6.7
Range	1–14

Cutaneous eruption occurrence to initiation of systemic treatment	*n* = 17	
Before diagnosis	3	18
Before treatment	8	47
During treatment	3	18
After treatment	2	12
Unknown	1	6

Latency of treatment for CLL/SLL (years)		
Mean	5	
Range	0–11	

Treatment of CLL/SLL		
Alkylating agents	7	41
PI3K inhibitor	1	6
CD20 mab	11	65
Purine analog	2	12
BH3‐mimetics	1	6
BMT	1	6
BTK inhibitor	8	47
None	2	12

CBC At time of skin biopsy	*n* = 16	
	Mean	Range
WBC (k/ul)	46.2	2.1–214.9
Granulocyte %	34.7	0–90
Lymphocyte %	57.7	7–100
Eosinophil %	2.7	0–10.6
HGB (g/dl)**	12.4	8.8–15.9

*Note*: The cutaneous eruptions were recorded in relation to the diagnosis of underlying CLL/SLL and systemic treatment initiation for CLL/SLL.

Abbreviations: BH3, BCL2 Homology 3; BMT, bone marrow transplant; BTK, burton tyrosine kinase; CLL, chronic lymphocytic leukemia; CBC, complete blood count; HGB, hemoglobin; IgHV, immunoglobulin heavy chain variable region; mab, monoclonal antibody; PPI3K, phosphoinositide 3‐kinase; SLL, small cell lymphocytic lymphoma; WBC, white blood count.

*One CLL patient developed Richter's transformation. **HGB was only available for 12 patients at the time of biopsy.

### 4.3 Histologic patterns (Table 3)

Twenty biopsies from 17 patients were reviewed, and histologic patterns were categorized. Overall, these biopsies were heterogeneous, demonstrating varied patterns and infiltrate characteristics. Spongiosis (70%) was the most common pattern seen in the epidermis. Nearly all biopsies had a prominent superficial and deep dermal eosinophilic infiltrate (85%); however, a robust lymphocytic infiltrate primarily composed of T‐cells (40% of biopsies) was also seen. The inflammatory infiltrates were best characterized as perivascular (90%) and periadnexal (70%) in most cases. Of the periadnexal infiltrates, 29% were folliculocentric with prominent folliculotropism and the remaining 41% were syringocentric or were mildly folliculocentric without significant folliculotropism. Inflammatory infiltrates were primarily limited to the papillary and reticular dermis but extended into the subcutis in 25% of cases. A granulomatous infiltrates resembling granuloma annulare (GA) were seen in 4 biopsies (20%).

**TABLE 3:** Summary of histological patterns and infiltrate characteristics for 20 biopsies of unknown cutaneous eruptions in patients with chronic lymphocytic leukemia (CLL)/SLL

Histopathological patterns	*n* = 20	(%)
Epidermis		
Spongiosis	14	70
Interface	5	25
Psoriasiform	1	5
Exocytosis	4	20
Erosion	3	15
Dermis		
Papillary edema	2	10
Flame Figures	2	10
Eosinophils	17	85
Lymphocytic	13	65
T cell infiltrate	8	40
B cell infiltrate	2	10
Granulomatous	4	20
Infiltrate characteristics		
Perivascular	18	90
Vasculitis	2	10
Periadnexal	14	70
Folliculotropism	5	25
Interstitial	6	30
Diffuse	8	40
Subcutaneous	5	25

*Note*: Predominant histological patterns from multiple biopsies demonstrated various characteristics determined by a panel of dermatopathologists.

**FIGURE 1:**
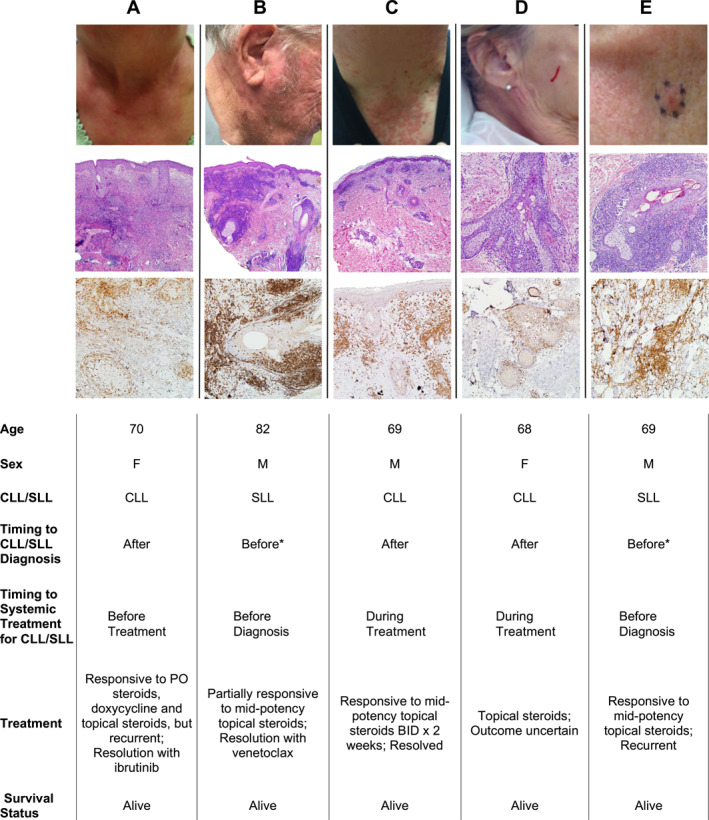
Clinical and histological findings for the five patients (A‐E) with a CD4+ T cell folliculocentric infiltrate. The top row corresponds to the clinical presentation at the time of cutaneous biopsy. Second and third rows present H&E and CD4 IHC, respectively (40x A‐C, second row; 100x D‐E second and A‐E third rows). *Cutaneous eruption preceded the diagnosis of chronic lymphocytic leukemia (CLL)/small lymphocytic lymphoma (SLL), and dermatologic evaluation prompted work‐up revealing underlying hematologic malignancy. CLL, chronic lymphocytic leukemia; SLL, small cell lymphocytic lymphoma.

*Folliculocentric hypersensitivity reactions:* Five out of 17 patients (29%) in our cohort demonstrated a predominately CD4+ T‐cell infiltrate characterized by a perivascular and periadnexal pattern that involved the follicular epithelium with exocytotic lymphocytes. Two of these patients demonstrated prominent follicular mucinosis. All patients had pruritic papules involving the face, neck, upper chest, and extremities, with three patients having CLL and two with SLL. Notably, the two patients with SLL had developed skin lesions before the diagnosis of SLL and dermatologic evaluation; work‐up prompted identification of the underlying hematopoietic malignancy. Four out of five patients received treatment for CLL/SLL, with complete resolution of cutaneous lesions being seen in two patients (50%). The prevalence of these folliculocentric infiltrates (*n* = 5) requiring biopsy in CLL/SLL patients in our cohort was 1%. Clinical and histological findings are summarized in Figure 1.

## 5 DISCUSSION

Hypersensitivity cutaneous eruptions in patients with CLL/SLL are rare, although the exact prevalence remains unknown. Previous reports have suggested that up to 6%–8% of patients with CLL may be affected.^4^, ^8^ Similarly, we demonstrated a prevalence for biopsy‐proven cutaneous hypersensitivity eruptions of 3.4% during our study period. These eruptions are likely underestimated due to their heterogeneous clinical and histological appearance. The nomenclature surrounding these cutaneous eruptions has been cumbersome but is evolving due to our increasing knowledge of the clinical and histological presentations. Robert Weed, in 1965, was the first to describe an exaggerated delayed hypersensitivity to mosquito bites in patients with CLL. His initial cohort represented eight out of 97 CLL patients with exaggerated insect bite reactions. Some individuals demonstrated similar manifestations on repeat exposure to subcutaneous mosquito antigen.^4^ This nomenclature later shifted to ‘insect bite‐like reactions’ as clinicians realized that these cutaneous eruptions occurred outside of arthropod exposure.^5^, ^8^, ^9^ Byrd *et al.* subsequently provided the basis for EDHM. Their criteria included: recurrent papulovesicular eruption with a superficial and deep eosinophilic infiltrate, history of hematologic malignancy, and the eruption cannot be explained by another pathologic process.^6^

Visseaux *et al.* proposed the term TCP‐BCM described in a cohort of 37 patients as a chronic pruritic papulovesicular eruption occurring commonly on the head/neck and in association with an underlying B‐cell malignancy, frequently CLL. These cutaneous eruptions demonstrated three main histological patterns: dermal CD4+ T‐cell infiltrate with variable eosinophils, folliculotropic CD4+ T‐cells resembling FMF, and infrequently epithelioid granulomatous infiltrates. Notably, folliculotropism was seen in 70% of the cases, resembling FMF histologically but lacked T‐cell receptor gene rearrangement.^7^ Similarly, in the present study we found 5 (29%) cases with significant CD4+ T‐cell folliculotropism resembling FMF, however, these cases did not present with the typical clinical findings of FMF (scaly patches, acneiform lesions, alopecia, or eyebrow involvement) nor progressed like FMF. Interestingly, there are few reports of mycosis fungoides (MF) and FMF being associated with B‐cell malignancies; however, distinction from pseudolymphomatous reactions is challenging, requiring clinicopathological correlation, immunohistochemistry, molecular studies, and longitudinal observation.^10^

The pathogenesis of hypersensitivity reactions in CLL/SLL and the exact role of the leukemic cells in this situation is uncertain. It is theorized that the underlying malignancy leads to an altered immunologic milieu with increased Th2 cytokine (interleukin‐4 & interleukin‐5), stimulating eosinophils.^2^, ^5^, ^9^ Malignant monoclonal B‐cells can be seen in up to 80% of these infiltrates but generally only represent a fraction of the cellular population (<1%‐20%).^2^, ^7^, ^8^ These leukemic cells are likely recruited bystanders from either exogenous or internal stimuli, contributing to an altered immune response.

It is hypothesized that the heterogeneity of cutaneous eruption may be related to the genetic landscape of the underlying malignancy, since molecular abnormalities are common in CLL, with over 80% of patients carrying at least one genetic aberration.^11^ This is the first study to investigate the correlation between the genetic and phenotypic risk profiling of CLL/SLL and the presence of cutaneous hypersensitivity. A 13q14 deletion is associated with a better prognosis, and in our cohort, it was the most common chromosomal aberration (*n* = 7, 50%). However, this is the most common FISH abnormality CLL with a prevalence ranging from 25% to 65% and may reflect the populational incidence.^12^, ^13^, ^14^ Poor prognostic molecular and cytogenetic findings, unmutated IgHV, trisomy 12, 11q22, and 17p13 deletions were not overly represented in our cohort, and rates appear comparable to previous reports in CLL without hypersensitivity eruptions.^14^, ^15^ Although our study is limited in number due to the rarity of the condition, it appears unlikely that the commonly tested specific molecular and cytogenetic aberrations are associated with paraneoplastic cutaneous eruptions.

The diverse clinical and histological spectrum of cutaneous hypersensitivity eruptions in CLL/SLL is further emphasized in this study. The most common clinical presentation included recurrent pruritic papular lesions above the waist; however, the clinical and histological morphologies were variable. A prominent eosinophilic infiltrate was seen in 85% of cases, which was accompanied by a lymphocytic infiltrate, principally T‐cells. A subset of patients (*n* = 5, 29%) had features of folliculocentric CD4+ T‐cells with a consistent clinical presentation; pruritic papular lesions on the head, neck, chest, and upper extremities. These clinical and histologic features share similarities to Visseaux's TCP‐BCM cohort.^7^ As TCP‐BCM is a relatively new designation, the incidence and prevalence are unknown. Our study's findings demonstrate a possible prevalence of a TCP‐BCM pattern or CD4+ T Cell folliculocentric infiltrates (*n* = 5) requiring a biopsy as approximately 1% in our cohort.

Additionally, we found a hypersensitivity reaction with a granulomatous infiltrate resembling GA in 20% of biopsies. This phenomenon has been previously described in CLL patients with Visseaux's cohort demonstrating GA‐like patterns in 14.5% of their biopsies.^7^, ^16^ Dermatopathologists should be aware of the presence of granulomatous infiltrates in CLL patients, which may be a diagnostic pitfall. Clinical correlation is required in these cases, and integration of clinical presentation and the presence of an eosinophil‐rich inflammation helps determine a cutaneous hypersensitivity with a secondary GA‐like pattern, such as in our cohort.

Although most of these unexplained hypersensitivity reactions occurred after a known diagnosis of CLL/SLL (70%), skin lesions preceded or occurred concurrently with the diagnosis of CLL/SLL in up to 30% of cases. Previous reports have demonstrated similar findings, with 22% of patients having insect bite‐like reactions before a known diagnosis of CLL.^17^ Due to the variability of the temporal relationship of the skin eruptions with the diagnosis of CLL, the importance of excluding a potential underlying leukemia/lymphoma in patients with an unexplained recurrent cutaneous eruption is underscored. Furthermore, two of the patients in our study had recalcitrant skin eruptions that preceded the diagnosis of SLL, which may present without CBC abnormalities, highlighting the importance of considering further ancillary studies and close clinical observation when a paraneoplastic reaction is a clinical possibility.

Topical steroids were partially effective in controlling pruritus and the development of lesions in all of our folliculocentric CD4+ T cell infiltrate patients (*n* = 5); however, only one patient had resolution with topical steroids. These findings are similar to the Visseaux *et al.* cohort, where topical steroids were effective in 82% of cases.^7^ Interestingly, two of four patients with severe refractory lesions demonstrated complete resolution after treatment for their underlying CLL/SLL [one with venetoclax (BH3‐mimetic) and the other with ibrutinib (BTK inhibitor)]. This treatment response of cutaneous hypersensitivity reactions is contrary to previous cohorts, which have not shown a relationship to cutaneous disease activity or course with treatment of the underlying hematologic malignancy.^5^, ^17^ However, it should be noted that these prior studies were conducted when conventional chemotherapy and anti‐CD20 monoclonal antibody therapy were the primary treatment for CLL/SLL. Our cohort reflects the changing dynamic in the management of CLL/SLL with more utilization of selective targeted or immunomodulating medications.

The present study is limited by its retrospective nature and small sample size; however, it supports findings reported by other authors and expands upon evidence for folliculocentric hypersensitivity reactions being a unique clinicopathologic entity.^2^, ^3^, ^5^, ^7^, ^8^ Our cohort centered on the diagnosis of CLL/SLL, but it should be noted that similar cutaneous eruptions have been reported in various other hematologic malignancies, HIV, and agammaglobulinemia.^18^, ^19^ Further studies are warranted to determine whether there might be related pathogenesis between these entities.

## 6 CONCLUSION

This study demonstrated the diverse clinical and pathologic patterns of cutaneous eruptions in the CLL/SLL population, and that folliculocentric CD4+ T cell infiltrates may represent a unique hypersensitivity pattern. Clinical correlation is imperative in making these diagnoses, and recalcitrant cutaneous hypersensitivity eruptions should be evaluated for an underlying malignancy. We report the novel findings that these cutaneous hypersensitivity reactions do not appear to be related to a high‐risk profile by cytogenetics and phenotyping. However, future research is needed to fully understand the complex immunologic milieu. Additionally, clinicians should consider systemic treatment of an underlying hematologic malignancy, as these malignancies may be contributing to the pathogenesis of the hypersensitivity reaction.

## CONFLICT OF INTEREST

The authors have no conflicts of interest to declare.

## AUTHOR CONTRIBUTIONS

**James Abbott**: Conceptualization (Equal); Data curation (Equal); Formal analysis (Equal); Funding acquisition (Equal); Investigation (Equal); Methodology (Equal); Project administration (Equal); Resources (Equal); Supervision, Visualization (Equal); Writing – original draft (Equal); Writing – review & editing (Equal). **Jessica Corean**: Conceptualization (Equal); Data curation (Equal); Writing – review & editing (Equal). **Ashley M. Snyder**: Data curation (Equal); Formal analysis (Equal); Writing – review & editing (Equal). **Scott R. Florell**: Methodology (Equal); Project administration (Equal); Resources (Equal); Supervision (Equal); Writing – review & editing (Equal). **Rodney Miles**: Conceptualization (Equal); Investigation (Equal); Methodology (Equal); Project administration (Equal); Resources (Equal); Supervision (Equal); Writing – review & editing (Equal). **Deborah Stephens**: Conceptualization (Equal); Methodology (Equal); Resources (Equal); Writing – review & editing (Equal). **David A. Wada**: Conceptualization (Equal); Data curation (Equal); Investigation (Equal); Methodology (Equal); Project administration (Equal); Resources (Equal); Supervision (Equal); Visualization (Equal); Writing – original draft (Equal); Writing – review & editing (Equal).

## ETHICS STATEMENT

This study has been approved by the University of Utah's Institutional Review Board (IRB_00076927) and received exempt status due to clinicopathologic correlation performed on retrospective material.

## Data Availability

Data is not readily available due to the retrospective nature and use of medical records containing PHI.
